# Managing Patients with Hypereosinophilic Syndrome: A Statement from the Italian Society of Allergy, Asthma, and Clinical Immunology (SIAAIC)

**DOI:** 10.3390/cells13141180

**Published:** 2024-07-11

**Authors:** Marco Caminati, Luisa Brussino, Matilde Carlucci, Palma Carlucci, Lucia Federica Carpagnano, Cristiano Caruso, Lorenzo Cosmi, Simona D’Amore, Stefano Del Giacco, Aikaterini Detoraki, Mario Di Gioacchino, Andrea Matucci, Ilaria Mormile, Francescopaolo Granata, Gabriella Guarnieri, Mauro Krampera, Matteo Maule, Eustachio Nettis, Stefania Nicola, Silvia Noviello, Fabrizio Pane, Cristina Papayannidis, Paola Parronchi, Girolamo Pelaia, Erminia Ridolo, Francesca Wanda Rossi, Gianenrico Senna, Massimo Triggiani, Angelo Vacca, Emanuele Vivarelli, Alessandra Vultaggio, Amato de Paulis

**Affiliations:** 1 Asthma Centre and Allergy Unit, Center for Hypereosinophilic Dysimmune Diseases, Department of Medicine, University of Verona, 37124 Verona, Italy; marco.caminati@univr.it (M.C.); matteo.maule@gmail.com (M.M.); gianenrico.senna@univr.it (G.S.); 2 SSDDU Immunologia Clinica ed Allergologia, AO Mauriziano, 10128 Turin, Italy; luisa.brussino@unito.it (L.B.); stefania.nicola@unito.it (S.N.); 3 Health Directorate, Verona Integrated University Hospital, 35134 Verona, Italy; matilde.carlucci@aovr.veneto.it; 4 Department of Emergency and Organ Transplantation, School of Allergology and Clinical Immunology, University of Bari Aldo Moro, 70126 Bari, Italy; palma.carlucci@uniba.it (P.C.); e.nettis@libero.it (E.N.); 5 Department of Diagnostics and Public Health, University of Verona, 37126 Verona, Italy; dr.fedecarpagnano@gmail.com; 6 Allergologia dell’Istituto di Clinica Medica del Policlinico Gemelli, Università Cattolica di Roma, 00168 Rome, Italy; cristiano.caruso@policlinicogemelli.it; 7 UOSD DH Internal Medicine and Digestive Disease, Fondazione Policlinico A Gemelli IRCCS, 00168 Rome, Italy; 8 Department Experimental and Clinical Medicine, University of Florence, 50121 Florence, Italy; lorenzo.cosmi@unifi.it (L.C.); paola.parronchi@unifi.it (P.P.); 9 Immunoallergology Unit, Careggi University Hospital, 50134 Florence, Italy; andrea.matucci@unifi.it; 10 Department of Precision and Regenerative Medicine and Ionian Area, UOC Medicina Interna “Guido Baccelli”, University of Bari Aldo Moro, Policlinico, 70126 Bari, Italy; simodamo@hotmail.it (S.D.); silvia.noviello88@gmail.com (S.N.); angelo.vacca@uniba.it (A.V.); 11 Department of Medical Sciences and Public Health, University of Cagliari, 09124 Cagliari, Italy; delgiac@gmail.com; 12 Division of Internal Medicine and Clinical Immunology, Department of Internal Medicine and Clinical Complexity University of Naples Federico II, 80138 Naples, Italy; kate.detoraki@gmail.com; 13 Center for Advanced Studies and Technology (CAST), G. D’Annunzio University of Chieti-Pescara, 66100 Chieti, Italy; digioacchino@me.com; 14 Institute of Clinical Immunotherapy and Advanced Biological Treatments, 66100 Pescara, Italy; 15 Department of Translational Medical Sciences, Federico II University, 80131 Naples, Italy; frapagra@hotmail.com (F.G.); francescawrossi@gmail.com (F.W.R.); depaulis@unina.it (A.d.P.); 16 Department of Engineering for Innovation Medicine, Section of Innovation Biomedicine, Hematology Area, University of Verona, 37129 Verona, Italy; gabriella.guarnieri@gmail.com (G.G.); mauro.krampera@univr.it (M.K.); 17 Department of Clinical Medicine and Surgery, University Federico II, 80138 Naples, Italy; fabrizio.pane@unina.it; 18 IRCCS Azienda Ospedaliero-Universitaria Di Bologna, Istituto Di Ematologia “Seràgnoli”, 40126 Bologna, Italy; cristina.papayannidis@unibo.it; 19 Immunology and Cell therapies Unit, University Hospital Careggi, 50134 Florence, Italy; 20 Department of Health Sciences, University “Magna Graecia” of Catanzaro, 88100 Catanzaro, Italy; pelaia@unicz.it; 21 Department of Clinical and Experimental Medicine, University of Parma, 43124 Parma, Italy; erminia.ridolo@unipr.it; 22 Center for Basic and Clinical Immunology Research (CISI), University of Naples Federico II, 80131 Naples, Italy; 23 WAO Center of Excellence, 80131 Naples, Italy; 24 Division of Allergy and Clinical Immunology, University of Salerno, 84084 Fisciano, Italy; mtriggiani@unisa.it; 25 Department of Biomedicine, Azienda Ospedaliero Universitaria Careggi, 50134 Florence, Italy; emanuele.vivarelli@gmail.com (E.V.); alessandra.vultaggio@unifi.it (A.V.)

**Keywords:** eosinophils, hypereosinophilia, hypereosinophilic syndrome, mepolizumab, management, network, precision medicine

## Abstract

Hypereosinophilic syndrome (HES) encompasses a heterogeneous and complex group of different subtypes within the wider group of hypereosinophilic disorders. Despite increasing research interest, several unmet needs in terms of disease identification, pathobiology, phenotyping, and personalized treatment remain to be addressed. Also, the prospective burden of non-malignant HES and, more in general, HE disorders is currently unknown. On a practical note, shortening the diagnostic delay and the time to an appropriate treatment approach probably represents the most urgent issue, even in light of the great impact of HES on the quality of life of affected patients. The present document represents the first action that the Italian Society of Allergy, Asthma, and Clinical Immunology (SIAAIC) has finalized within a wider project aiming to establish a collaborative national network on HES (InHES—Italian Network on HES) for patients and physicians. The first step of the project could not but focus on defining a common language as well as sharing with all of the medical community an update on the most recent advances in the field. In fact, the existing literature has been carefully reviewed in order to critically integrate the different views on the topic and derive practical recommendations on disease identification and treatment approaches.

## 1. Introduction

Human eosinophils are terminally differentiated leukocytes characterized by cytoplasmatic granules containing biologically active molecules, including eosinophil peroxidase, eosinophil cationic protein, major basic protein, and several cytokines, such as transforming growth factor (TGF)-ß [1]. Eosinophils are produced in the bone marrow following the development of multipotent hematopoietic stem cells into eosinophil-committed progenitors (EoPs), which then differentiate into mature eosinophils [2]. Once mature, eosinophils leave the bone marrow and enter the bloodstream, where they circulate for about one day before migrating to tissues such as the mucosa of the gastrointestinal (GI) tract (from stomach to colon), thymus, spleen, lymph nodes, and uterus [1,2,3,4]. Human eosinophils are not typically found in other healthy tissues and organs [1].

In terms of function, eosinophils largely contribute to the host’s defense mechanisms against external pathogens [2], but more recently, they have been linked to the regulation of tissue remodeling and fibrosis as well as the regulation of other immune responses [2].

Eosinophilia is defined by increased eosinophils in the peripheral blood and is observed in several inflammatory and reactive conditions [5]. Peripheral blood absolute eosinophil count (AEC) normal value ranges between 0.05 and 0.5 × 10^9^/L, while reference values for mature eosinophils in bone marrow aspirates are between 1% and 6% [1,6,7,8]. According to the classification of eosinophilic disorders proposed in 2011 by the International Cooperative Working Group on Eosinophil Disorders (ICOG-EO) and revised in 2022, blood eosinophilia is defined by an AEC above 0.5 × 10^9^/L, while hypereosinophilia (HE) necessitates an AEC of ≥1.5 × 10^9^/L [1,6]. The AEC increase can be classified as mild eosinophilia (0.5–1.49 × 10^9^/L), moderate HE 1.5–5.0 × 10^9^/L, and severe HE (>5.0 × 10^9^/L) [1,9]. As transient causes can also cause eosinophilia, another classifying criterion is the persistence of the increase in eosinophil count. Indeed, eosinophilia can be episodic, transient, or persistent [6]. The temporal interval to define eosinophilia as “persistent” has been widely discussed. In the first definition comprised in Chusid criteria [10], an AEC persistently increased (i.e., longer than 6 months) above 1.5 × 10^9^/L was required. Then, a 4-week interval was proposed [11]. Finally, the latest Working Conference on Eosinophil Disorders and Syndromes (Vienna, 24–26 September 2021) defined “persistent” HE as the evidence of AEC ≥ 1.5 × 10^9^/L recorded at least twice in a minimum time interval of two weeks [6]. This choice was based on the availability of more rapid diagnostic tests and a time-saving strategy for cases with potentially rapid-onset eosinophil-related organ dysfunction [12]. Identifying a possible underlying cause of transient HE and the HES subtype is crucial in order to choose the most suitable treatment and schedule an appropriate follow-up. In this view, the second determination is pivotal. However, treating the patients or adopting a “watch and wait” strategy after the first determination of HE should always be based on the patient’s symptoms and the potential eosinophil-related organ involvement. Hence, the treatment approach should be evaluated for each patient, scheduling an accurate follow-up for monitoring symptom and organ involvement in untreated patients with HE. 

Criteria for tissue HE has been defined as the presence of one or more of the following conditions documenting the eosinophilic infiltrates or the presence of their products: (i) eosinophils represent more than 20% of all the nucleated cells in bone marrow sections; (ii) an extensive (massive) tissue infiltration by eosinophils compared with “normal physiologic ranges” is documented by a pathologist; (iii) immunostaining reveals the marked extracellular deposition of eosinophil-derived proteins (e.g., eMBP1 or EPX) [1,6,12].

Indeed, persistent HE may be associated with eosinophil infiltration into tissues, thus leading to tissue and organ damage caused mainly by the release of eosinophil effector molecules [1,13,14]. HE-related end-organ damage can be associated with significant morbidity and mortality [1,15]. 

It should also be noted that not all the patients with HE present with end-organ manifestation, and can be classified as affected by the HE of unknown significance (HEus) [16].

HE with organ involvement caused by marked eosinophil infiltration is defined as hypereosinophilic syndrome (HES) [17]. The concept of HES was introduced in the late 1960s [17]. Today, HES refers to a heterogeneous group of rare diseases whose diagnosis and treatment remain challenging [17]. Thanks to advances in the identification of the genetic alterations underlying HE, the identification of novel variants and the classification of these conditions are rapidly evolving and require regular updating [5,6,18]. These advances are also reflected in the increasing availability of treatments, especially targeted therapies.

This article aims to provide an overview of the updated definitions and classifications of HE and HES and the current management of these rare diseases, from the diagnosis and assessment of organ involvement to treatment selection. Recommendations for the management of patients with HES and suggestions for implementing a multidisciplinary approach have been included. The focus is on primary HES and idiopathic HES. These recommendations and suggestions are based on a review of the recent literature, existing relevant guidelines, and the authors’ clinical experience. The Italian Society of Allergology, Asthma, and Clinical Immunology (SIAAIC) has endorsed these recommendations.

## 2. Definition and Classification of Hypereosinophilia and Related Syndromes

A variety of different classifications of eosinophil disorders have been proposed over the years (Table 1) [18]. 

A general distinction between primary (or clonal) HE, secondary (or reactive), or idiopathic when the causes remain undetermined is proposed by most of the authors (Table 1). Of note, secondary hypereosinophilia is a relatively common disorder, whereas the primary and idiopathic subtypes are rare [17]. Among the reactive HES, many allergic conditions; infections; endocrine disorders; some hematological disorders, including leukemia and lymphomas; solid neoplasms; and immunodeficiencies are responsible for the disease [19].

A further recurrent approach identifies within the non-reactive forms the following subtypes: myeloproliferative variant (M-HES), lymphocytic variant (L-HES), familial, undefined, overlap, and associated [19,23]. The neoplastic forms of HES are sustained by hematological malignancy or bone marrow abnormalities, typical for myeloproliferative disorders, or by a clonal population of activated T-lymphocytes [19]. Familial HES is an extremely rare condition, mainly linked to a mutation in the gene located on chromosome 5q31-33. On the other hand, HEus is defined in this case as a benign form of eosinophilic disease, with long-time persistent blood hypereosinophilia, but no signs of end-organ dysfunction attributable to eosinophilia. 

Regarding the distinction between overlap and associated HES, the first subtype includes conditions that are associated with HE and single organ damage, but they have a completely different pathogenesis, including eosinophilic granulomatosis with polyangiitis (EGPA), immunoglobulin (Ig)G4-related disease, allergic bronchopulmonary aspergillosis (ABPA), and some eosinophilic disorders of the digestive tract (EGID). Associated HES refers to peripheral hypereosinophilia in association with a defined diagnosis, such as systemic mastocytosis, infections, inflammatory bowel disease, systemic vasculitis, and other autoimmune diseases. A partial overlap among the reactive and associated HES definitions has to be noted. A slightly different perspective, although no more included in recent classifications, focused on the pathogenic role of eosinophils by identifying two major forms only: intrinsic disorders (e.g., caused by neoplastic eosinophils) and extrinsic disorders (caused by the action of other cells) [22].

More recently, the World Health Organization (WHO) has released its definition of eosinophilic disorders. According to that classification, the diagnosis of HES_I_ may be considered provisional until the cause of eosinophilia is ascertained [5]. It should be noted that, with an increasing number of molecular markers of neoplasia, fewer patients are being diagnosed with HES_I_, but the percentage is still too large.

To refine the HE_N_ category and identify the underlying neoplasm, the IGOC-EO classification relies on the WHO and the International Consensus Classification (ICC) classifications of myeloid neoplasms [5,11,24]. These neoplasms include “chronic eosinophilic leukemia, not otherwise specified” (CEL NOS), which belongs to the myeloproliferative neoplasms (MPNs) category, and a recently introduced category named “myeloid/lymphoid neoplasms with eosinophilia and tyrosine kinase gene fusion” (MLN-TK) [11,24]. These neoplasms are caused by the rearrangements of the genes encoding specific tyrosine kinases, leading to fusion products wherein the kinase is constitutively active. This results in altered cell signaling, causing changes in proliferation and survival [11]. MLN-TK is defined by different genetic anomalies, specifically the rearrangements of platelet-derived growth factor receptor alpha (PDGFRA), PDGFR beta (PDGFRB), fibroblast growth factor receptor (FGFR)1, Janus kinase (JAK)2, FLT3, and PCM1-JAK2 fusion [5,18].

A new attempt at HES classification has been recently proposed by Valent and co-authors [6], including many significant modifications. As a matter of fact, the authors included the overlap forms of HES as a special form of reactive HES, thus blinding the underlying pathogenetic mechanism. Moreover, lymphocytic HES has also been considered among the reactive forms of HES, despite the fact that the underlying cause is a clonal disorder, albeit of lymphoid origin. Lastly, the neoplastic HES classification was also changed, with the clonal myeloid and lymphocytic HES grouped with the solid organ HES, even though the HE was sustained by a cytokine-driven clonal proliferation in the first case. In contrast, in the latter, the HE is a reactive form, associated with the inflammatory stimulus of the malignancy [6].

According to the aforementioned classification, the HE variants and related syndromes are defined and abbreviated as follows: familial (hereditary) HE (HEFA), neoplastic (clonal or primary) HE (HEN), reactive (secondary) HE (HER), the HE of undetermined significance (HEUS), and idiopathic HES (HESI) [6]. HER is the most common variant, in which eosinophilia is driven by cytokine overproduction, whereas in HEN, eosinophilia is caused by neoplastic eosinophil clones [6].

**Figure 1:**
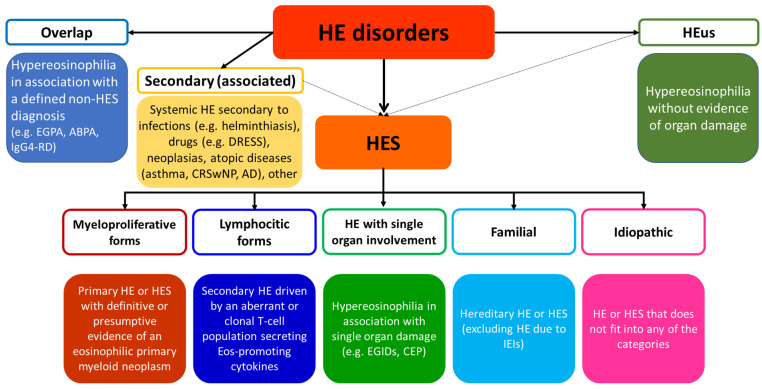
SIAAIC proposal for a revisited classification of hypereosinophilic disorders. ABPA: allergic bronchopulmonary aspergillosis; AD: atopic dermatitis; CEP: chronic eosinophilic pneumonia; CRwNP: chronic rhinosinusitis with nasal polyps; DRESS: drug rash with eosinophilia and systemic symptoms; EGIDs: eosinophilic disorders of the digestive tract; EGPA: eosinophilic granulomatosis with polyangiitis; HE: hypereosinophilia; HES: hypereosinophilic syndrome; HEus: hypereosinophilia of unknown significance; IEI: inborn errors of immunity.

Figure 1 summarizes a revised classification proposed by the authors. The main background is represented by the aforementioned work by Valent et al., still with some differences. In fact, the purpose of the SIAAIC task force is to support clinicians with different backgrounds in their approach to HES by providing an easy-to-understand classification which mainly reflects the clinical presentation, underlying mechanisms, and the potential burden of different hypereosinophilic disorders and HES subtypes. The following are the main points which distinguish the newly proposed classification from Valent’s and other works: -HE disorders and HES subtypes are presented separately, still valuing the potential evolution of hypereosinophilic disorders towards hypereosinophilic syndromes; -overlap and reactive hypereosinophilic disorders have been separately included and distinguished by HES subtypes. In fact, overlap form refers to hypereosinophilia in association with a defined non-HES condition, expressing an immunological background different from HES, still with chronic and potential multiple organ burden; on the other side, secondary HE disorders develop as a reaction to a specific trigger (allergens or pathogens) with limited, usually single-organ involvement; -in order to highlight its clonal background and potential evolution, lymphocytic subtype has been included separately from the reactive forms of HES. 

## 3. Diagnosis

**Figure 2:**
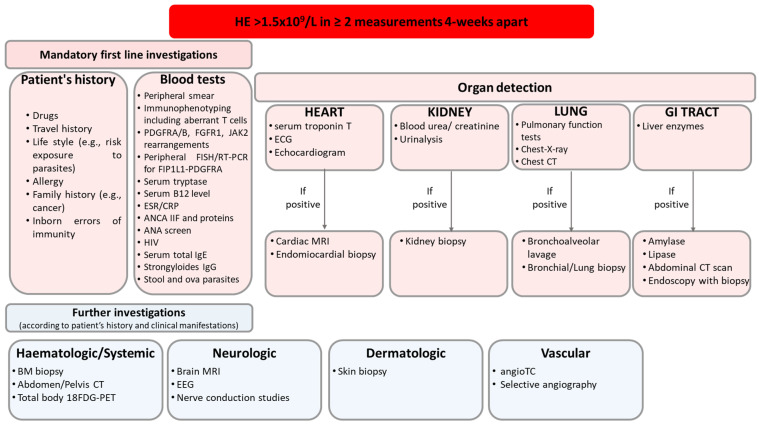
Mandatory and optional assessment following the detection of blood hypereosinophilia suggestive of HES. ANA, antinuclear antibody; ANCA, anti-neutrophil cytoplasmic antibodies; CRP, C-reactive protein; CT, computed tomography; ECG, electrocardiogram; ESR, erythrocyte sedimentation rate; FGFR1, fibroblast growth factor receptor 1; FIP1L1, factor interacting with PAPOLA and CPSF1; JAK2, Janus kinase 2; MRI, magnetic resonance imaging; PDGFRA, platelet-derived growth factor receptor alpha; PET, positron emission tomography.

The symptoms and disease severity can vary considerably among patients with eosinophilia [17]. Interestingly, the clinical manifestation of HE can be identical regardless of its cause [25]. In the first step of the diagnostic work-up, the presence of HE is confirmed. Then, the investigation focuses on determining the etiology of HE, particularly whether an underlying disease is contributing [6]. The initial assessment includes a consideration of family, as familial HE (HE_FA_) is possible, although rare (the genetic transmission of HE has been clearly demonstrated in a very small number of families only) [2,6]. The presence or absence of a reactive process is established based on clinical and laboratory features [6]. If HE_R_ is confirmed, efforts are directed towards identifying the specific disease process causing HE (inflammation, infection, tumor, and drug hypersensitivity). Indeed, HE_R_/HES_R_ is best managed by treating the underlying condition [20]. When primary clonal HE (HE_N_) is detected, it is crucial to determine the underlying hematological neoplasm according to the latest WHO and ICC classifications [5,6,11,24]. The patients without an underlying reactive condition and no sign of clonality are provisionally diagnosed with HE_US_ [6]. These patients must be carefully monitored over time, especially for the presence of organ involvement [6]. Finally, the investigation is extended to assess organ involvement, and the patients with HE-related organ damage are diagnosed with HES (Figure 2).

### 3.1. Exclusion of Hypereosinophilia Secondary to Other Conditions

The secondary causes of HE are wide-ranging and comprehensively reviewed elsewhere [5,6,25,26,27]. These causes should be considered in all the patients presenting with HE as they are often treatable by eliminating the causative agent or by treating the underlying disease [3].

Eosinophilia is generally uncommon in bacterial and viral diseases, with the exception of tuberculosis and human immunodeficiency virus (HIV) infection, where mild eosinophilia can occur, especially in association with parasitic and fungal illnesses. HE is typically associated with infections by parasitic worms (helminths), but can also be induced by fungal infections [28]. Infections with *Strongyloides stercoralis*, a soil-transmitted helminth, are common worldwide and can cause abdominal discomfort or remain completely asymptomatic in the chronic stage [28]. Parasitic myositis caused by *Trichinella* spp. and *Sarcocystis* spp. infections are associated with severe HE; hookworms and *Toxocara Canis* spp. also lead to severe HE [28]. Lung infections caused by the fungus *Coccidioides immitis*, crayfish-borne *Paragonimus* spp. (a flatworm), and *Dirofilaria immitis* (heartworm) may lead to significant eosinophilia [28]. The crusted scabies and ruptured cysts of *Echinococcus* spp. (a tapeworm) rarely provoke eosinophilia [28]. *Anisakis simplex* (a roundworm), usually ingested with raw fish, can cause abdominal pain and urticaria [28]. In this last case, although possible, peripheral eosinophilia is not present in the majority of gastric or intestinal anisakiasis [28,29,30,31]. In addition, an increased number of eosinophils in ascites has been described in a case series of patients diagnosed with intestinal anisakidosis [32]. Finally, eosinophilic pleural effusion and eosinophilia have been reported in a patient with pulmonary anisakiasis [33].

Drug hypersensitivity is a frequent cause of HE [34,35,36]. The manifestations of drug-induced HE are highly variable and can involve a single organ or multiple systems. Overall, asymptomatic eosinophilia is typically associated with the use of antibiotics, including beta-lactams and quinolones [35]. Drug-induced hypereosinophilia clinically presents as a single organ damage, such as interstitial nephritis. Maculopapular rash is observed with several drug classes, such as beta-lactam antibiotics, sulfonamide antimicrobials, allopurinol, anti-epileptic drugs, diuretics, and nonsteroidal anti-inflammatory drugs (NSAIDs) [37]. Drug-induced eosinophilia accompanied by systemic symptoms such as fever, hepatitis, and morbilliform rash has been defined as DRESS and can be observed, among others, with the use of anti-epileptics, beta-lactams, quinolones, NSAIDs, antituberculosis medications, and allopurinol [35,36,38].

Summary statements

- Several infectious diseases and some drugs can cause secondary hypereosinophilia.
- Helminths are the most associated parasites with hypereosinophilia.
- Several drugs can cause hypereosinophilia in some cases, leading to a potentially life-threatening delayed drug-related hypersensitivity reaction (DRESS).

### 3.2. Neoplastic Hypereosinophilia 

Several hematological malignancies can lead to HE and serve as a cause of secondary HE/HES, characterized by an expansion of the eosinophil lineage due to interleukin (IL)-5 overproduction [2,15]. These conditions normally lack the genome rearrangements detected in primary HE/HES (see Section 3.2) [5], and can be classified into hematologic malignancies leading to clonal eosinophilia and hematologic malignancies leading to polyclonal eosinophilia [25,39].

The first category includes acute myelomonocytic leukemia with eosinophilia and chronic myeloid leukemia (CML) [40,41]. Acute myelomonocytic leukemia with eosinophilia, formerly known as FAB AML M4Eo, is a rare form of acute myeloid leukemia (AML) which is characterized by chromosome 16 abnormalities [(inv(16)(p13.1q22) or t(16;16)(p13.1;q22)/CBFB::MYH11] [11,24]. Its symptoms are attributed not to an increase in immature eosinophils in the blood but to pancytopenia, resulting in anemia, infections, and bleeding [42]. Chronic myeloid leukemia (CML), a myeloproliferative neoplasm characterized by the presence of the Philadelphia chromosome and constitutively active BCR-ABL1 tyrosine kinase [11,24], is linked to an increased number of circulating neutrophils, myeloid progenitors, basophils, and eosinophils. However, eosinophils rarely prevail over neutrophils and are not associated with specific symptoms [11,24,40,43]. 

Hematological malignancies leading to polyclonal eosinophilia include T-cell lymphomas, B-cell lymphomas, B-cell acute lymphoblastic leukemia (B-ALL), and systemic mastocytosis (SM) [2]. Even after chemotherapy, T-cell lymphoblastic lymphoma, adult T-cell leukemia/lymphoma, and angioimmunoblastic T-cell lymphoma may be associated with secondary and polyclonal peripheral eosinophilia in up to 30% of the patients [44]. Both the variants of cutaneous T-cell lymphoma (e.g., Sezary syndrome and mycosis fungoides) can be associated with increased serum IgE and eosinophilia in up to 20% of the patients [45]. It is noteworthy that determining T-cell receptor clonality when eosinophilia is found may be useful in diagnosing unknown T-cell lymphomas [44,46,47]. B-cell lymphomas, whether Hodgkin or non-Hodgkin, are commonly associated with polyclonal tissue eosinophilia, while peripheral blood eosinophilia seldom is usually mild [48]. Specifically, in the context of B-ALL, the occurrence of polyclonal peripheral blood eosinophilia is rare and mainly related to the t(5;14) translocation. Eosinophilia may precede the development of clinical manifestations [49,50]. SM is caused by clonal mast cell proliferation carrying the D816V mutation of the *c-KIT* gene. It is characterized by a systemic or allergic-like involvement of the skin (urticaria pigmentosa), spleen, liver, lymph nodes, bone, and bone marrow [51]. Secondary and polyclonal peripheral blood eosinophilia may occur in up to 20% of the patients [52].

Solid cancers may also be associated with polyclonal eosinophilia as a kind of epiphenomenon and include several adenocarcinomas of the lung and GI tract (stomach and large bowel), as well as squamous carcinomas (skin, nasopharynx, bladder, cervix, vagina, penis, and breast) [53]. Polyclonal eosinophilia often precedes the clinical manifestation of these solid cancers [39].

### 3.3. Molecular Diagnosis of the Underlying Hematologic Neoplasm

In the case of persistent HE, once secondary causes are ruled out, the diagnostic work-up should focus on determining whether a clonal bone marrow disease is present. Several clinical (e.g., hepatomegaly and splenomegaly) and laboratory (e.g., cytopenia, thrombocytosis, polycythemia, monocytosis, basophilia, and elevated serum vitamin B12 and/or tryptase) characteristics, along with the inability to normalize blood eosinophil count with systemic corticosteroids, suggest the clonal nature of HE [5,6].

According to the latest recommendations on the classification and management of eosinophilic disorders, three entities should be considered based on the 2022 WHO and ICC classifications of myeloid neoplasms [11,24]: MLN-TK (including *PDGFRA*, *PDGFRB*, *FGFR1*, *JAK2*, *FLT3*, *PCM1-JAK2* fusion, *ETV6-ABL1* fusion, and other defined tyrosine kinase fusions) [40,54,55,56,57,58], myeloid malignancies associated eosinophilia [40], and CEL NOS [5,6]. Given that the majority of MLN-TK cases show *PDGFRA* rearrangements, the search for the *FIP1L1-PDGFRA* fusion should be prioritized. This search can be carried out using nested reverse transcription polymerase chain reaction (PCR) or real-time quantitative PCR in peripheral blood. PCR-based techniques are more sensitive than fluorescence in situ hybridization (FISH) when it comes to detecting the CHIC2 4q12 deletion (a surrogate for the *FIP1L1-PDGFRA* fusion) [59]. However, FISH may be useful in the very rare event of an atypical translocation, where PCR could potentially produce false-negative results. If the *FIP1L1-PDGFRA* fusion is absent, conventional karyotyping with FISH on blood samples or bone marrow aspirate is recommended. FISH can also detect rearrangements involving: *PDGFRB* [including the *ETV6-PDGFRB* fusion with chromosomal translocation t(5;12)(q32;p13)], *JAK2* [including the *PCM1-JAK2* fusion associated with t(8;9)(p22;p24)], *FLT3* [including the *ETV6-FLT3* fusion with t(12;13)(p13;q12)], and *FGFR1* (8p11 myeloproliferative syndrome) [60,61,62]. Identifying these molecular rearrangements is crucial because of their responsiveness to available target therapies, including the tyrosine kinase inhibitor imatinib (*PDGFRA* and *PDGFRB*), FGFR1 inhibitors (within clinical trials, followed by allogeneic hematopoietic stem cell transplant), and JAK2 inhibitors.

Myeloid malignancies linked with eosinophilia include myelodysplastic neoplasms (MDS), MPNs, MDS/MPNs, AML, and SM [40]. A diagnosis of any of these malignancies should be suspected upon the observation of hematologic abnormalities and clinical indicators. Furthermore, when polycythemia and/or essential thrombocytosis coincide with HE, testing for the JAK2 V617F mutation, CALR, and MPL mutations is recommended. The evaluation of the *BCR-ABL1* fusion is also necessary in the case of neutrophilia or basophilia associated with HE. 

CEL NOS is diagnosed when screening for eosinophilia related to MLN-TK is negative and there is cytogenetic, molecular, and/or morphologic evidence of an eosinophilic myeloid malignancy that cannot be classified otherwise. CEL NOS may show nonspecific clonal cytogenetics, molecular abnormalities, or an increased blast cell count (>2% in peripheral blood or >5% in bone marrow, but <20% in both compartments) [18,63].

It should be noted that next-generation sequencing (NGS) enables the simultaneous analysis of multiple genomic alterations and has proven valuable in other oncologic settings. However, NGS is still being investigated for diagnosing eosinophilic neoplasms and is not widely used in clinical practice [63,64,65]. Therefore, the pathogenic relevance of NGS findings still requires careful assessment. 

Lymphocyte phenotyping by flow cytometry on peripheral blood or bone marrow is also part of the molecular diagnostic work-up of HES. In fact, L-HES may be associated with clonal and/or aberrant T-cell populations. A clonal T-cell receptor (TCR) gene rearrangement can also be found in that subtype. A common pattern recurring in L-HES patients is the absence of CD3 (e.g., CD3-CD4+), a preserved T-cell receptor complex, or double negative, immature T-cells (e.g., CD3+CD4-CD8-). However, different profiles have been detected, including CD3+ CD4+ CD7−, CD3+ CD4− CD8− TCRαβ+, and loss of CD7, and/or CD27+ [66,67]. Around one out of two patients affected by L-HES demonstrates the clonal rearrangement of T-cell receptor genes, which actually recurs in various HES subtypes and in 17–27% of the patients with unexplained eosinophilia [46,47].

Summary statements

- Once secondary causes are ruled out, whether a clonal bone marrow disease is present needs to be excluded.
- PCR/real-time quantitative PCR, or FISH, is helpful in assessing FIP1L1-PDGFRA gene fusion and less common mutations and rearrangements, including PDGFRB, FGFR1, and FLT3 JAK 2.
- Flow cytometry lymphocyte phenotyping identifies clonal and/or aberrant T-cell populations, which may be associated with L-HES.

### 3.4. Assessment of Systemic Involvement

In patients with HES, multiple organs are frequently affected [68,69]. The typical features of HES-related organ damage include fibrosis, thrombosis, cutaneous (skin or mucosa) erythema, edema/angioedema, blisters, ulceration, or eczema, pulmonary manifestations, GI involvement, peripheral or central neuropathy with neurological deficits, and eosinophilic vasculitis [6,68]. According to the results of a retrospective analysis of 188 patients diagnosed with HES, the skin was the most frequently affected organ at presentation (37%), followed by the lung (25%), GI tract (14%), heart (5%), and central nervous system (4%) [70].

Once the secondary causes of eosinophilia have been ruled out, patients with persistent HE should be assessed, irrespective of the symptoms’ presence. This evaluation should include both imaging and histologic examinations. In patients with HES, lung function tests with diffusion capacity for carbon monoxide (DLCO) are the first step for lung assessment. The high-resolution computed tomography (HRCT) of the chest can show patchy ground-glass opacities and consolidation areas, but nodular opacities can also be observed [71]. Lung biopsy may reveal eosinophilic infiltrates in airway walls and alveolar septa, along with the collection of eosinophils and macrophages within air spaces [71]. The characteristics of cardiac involvement due to HES include mural thrombi, endomyocardial fibrosis, and restrictive cardiomyopathy with congestive heart failure [72]. Cardiac assessment should include a heart ultrasound as a routine examination due to the lack of symptoms in the early phases of heart involvement. On the other hand, magnetic resonance imaging (MRI) allows the detection of subtle perturbations and discrimination between inflammatory and fibrotic stages [72]. Endomyocardial biopsy may be useful if blood eosinophilia is not marked, but should always be performed after carefully evaluating the risk–benefit balance [72].

HES affecting the GI system can manifest as an isolated GI disorder or may be accompanied by HES-related disorders affecting other systems. Tissue eosinophilia, evaluated via upper or lower endoscopy, is considered abnormal when the peak eosinophil count is ≥15 eosinophils per high power field (EOS/HPF) in the esophagus, ≥30 EOS/HPF in the stomach or small bowel, and ≥60 EOS/HPF in the large bowel [73].

Summary statements

- HES is, by definition, a systemic condition and is related to the pathobiological burden of eosinophils, including coagulation impairment, inflammation, and tissue damage.
- Organ-specific involvement has to be explored and specifically addressed.

## 4. Organ-Specific Management

### 4.1. Respiratory System

Although HES typically affects multiple organs, HE-related damage can often be restricted solely to the respiratory tract [74]. Eosinophilic infiltration can involve both the upper and lower airways, extending to alveolar spaces and the lung interstitium [4,75,76]. Common upper and lower airways chronic manifestations, including chronic rhinosinusitis with nasal polyposis (CRSwNP) [77] and asthma, which actually express local eosinophilic infiltration, if associated with blood eosinophilia can be considered part of HES manifestations [14,78]. 

The other relevant pulmonary manifestations of HES include chronic eosinophilic pneumonia, featured by the accumulation of eosinophils within alveoli and lung interstitium [75,76]. Eosinophilic infiltration in extensive lung areas impairs gas exchange, causing hypoxemia and shortness of breath. Different imaging techniques, such as standard chest X-ray radiography and computed tomography (CT) scans, allow the identification of alveolar eosinophilia as bilateral lung infiltrates. In patients with HES associated with chronic eosinophilic pneumonia, elevated eosinophil count can be detected in bronchoalveolar lavage fluid (BALF) [68].

Moreover, patients with HES and pulmonary involvement may experience dyspnea arising from eosinophilic pleural effusions caused by the accumulation of eosinophils within the pleural space [79]. Abundant eosinophilic pleural effusions are characterized by abnormal gas exchange due to extraparenchymal lung compression [68,79]. Concerning the etiology, eosinophilic pleural effusions can be idiopathic, or caused by parasitic infections, drug reactions, and malignancies [68].

The diagnosis of airway involvement is challenging and requires a combination of clinical and imaging features along with laboratory findings. The absolute eosinophil count in peripheral blood and the eosinophil percentage in BALF are crucial for evaluating the various eosinophilic lung diseases. Lung functional tests, including spirometry and DLCO, are relevant for diagnosing obstructive diseases. Although chest radiography is the primary imaging technique for suspected pulmonary eosinophilic disorders, CT scans can reveal characteristic pulmonary patterns, nodules, and subtle parenchymal abnormalities [80].

Summary statements

- Respiratory manifestations are common in HES patients.
- Eosinophilic infiltration can involve upper and lower airways, as well as alveolar spaces and the lung interstitium.
- HRCT scan and BALF, as well as lung function assessment, are helpful tools in exploring respiratory involvement in HES.

### 4.2. Skin

The skin is one of the most frequently involved organ systems in HES, with over 50% of the patients developing cutaneous and/or mucous membrane manifestations [81,82]. Skin involvement occurs more frequently in the lymphocytic variant of HES and may represent the sole clinical feature [69].

**Figure 3:**
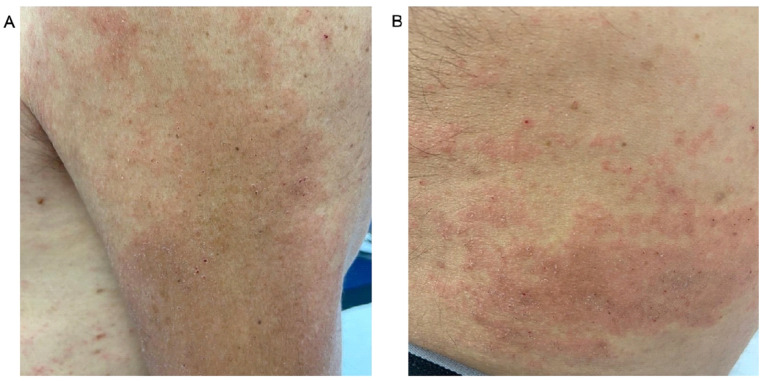
Skin manifestations of hypereosinophilic syndrome. Well-delineated maculopapules, eczematous lesions, and excoriated eruptions localized at the upper limbs (**A**), trunk, and abdominal skin (**B**).

Dermatologic manifestations are the most common presenting clinical signs of HES [83], and are typically heterogeneous [70] and include diffuse or localized pruritus, urticarial lesions [84], eczematous and/or itchy papulonodular lesions similar to atopic dermatitis [85,86], and prurigo nodularis [87] (Figure 3). 

Other, uncommon, dermatologic findings in HES are the recurrent attacks of facial angioedema [88,89], palpable purpura associated with necrotic–ulcerative lesions similar to vasculitic lesions [90], ulcerated plaques [91], and erythroderma [92]. Patients with HES may also develop erythema annulare centrifugum and livedo reticularis [93].

Necrotic lesions involving the oral cavity have also been reported, in particular in LHES [94] and M-HES [95]. They are particularly painful and difficult to treat and may be misdiagnosed as Bechet’s syndrome. The ulcers may be refractory to conventional HES treatments but appear to respond to the treatment with imatinib [69,93].

In HES, the histopathology of cutaneous lesions is nonspecific [96]. Urticarial lesions show a variable pattern of cell infiltration (lymphocytes, eosinophils, and neutrophils) in a perivascular distribution, as in common urticaria [96]. Papules or plaques exhibit spongiosis in addition to dermal infiltrates containing at least a few intact eosinophils. The thrombosis of dermal blood vessels has been observed in biopsy specimens from retiform purpura and necrotic skin lesions. The biopsy specimens of mucosal ulcers from patients with HES show the extensive deposition of eosinophilic granule proteins in the absence of morphologically identifiable intact eosinophils [97].

HES is a complex disease, and the diagnosis of its skin manifestations is challenging because mucocutaneous lesions are highly variable and similar to those of several other dermatologic conditions. The presence of peripheral and tissue eosinophilia makes differential diagnosis quite broad, ranging from drug reactions and allergies to proliferative hematologic disorders, vasculitic disorders, infectious diseases, and other diseases [98,99].

Summary statements

- The skin is one of the organ systems most frequently involved in HES.
- Dermatologic manifestations in HES represent the most common presenting clinical signs.
- Skin manifestations are highly variable and may be mistaken for several dermatologic conditions.

### 4.3. Gastrointestinal System

GI involvement is a common clinical manifestation of HES [70]. GI disease can be the only manifestation of HES or be accompanied by the dysfunction of other organs [70]. Isolated HE-related GI disease appears to overlap with primary EGIDs, which may represent the onset of a subsequent multisystemic form of HES [100]. To support this concept and reduce delays in diagnosis, a further clinical entity defined as “organ-restricted HES” has been recently proposed [100]. This entity is defined by single tissue/organ eosinophilic infiltration, without the hematologic criteria of blood HE. In this way, potential HES is not overlooked, and patients receive similar management as those diagnosed with bona fide HES [6]. Nevertheless, in both multisystemic and GI-restricted HES, other conditions that may cause GI damage must be excluded. For this reason, an attentive differential diagnosis should be made, excluding reactive conditions (e.g., parasitosis, chronic inflammatory bowel disorders, allergies, and drug reactions), paraneoplastic conditions, and HE-related SM [20].

Regarding clinical presentation, two main categories can be defined: eosinophilic esophagitis (EoE) and non-esophagitis eosinophil GI disease, with the latter being divided into eosinophilic gastritis (EoG), enteritis (EoN), and colitis (EoC), which may occur in different combinations [101]. Of note, EoE is rarely associated with blood hypereosinophilia and less frequently a part of HES manifestations compared to non-esophagitis eosinophil GI diseases. However, EoE symptoms deserve to be investigated when HES is suspected and, in contrast, blood eosinophil count should be evaluated in EoE patients.

EoE involves a dysregulated immune response resulting from a complex interplay between genetic and environmental factors [102]. Indeed, genes such as TSLP, calpain-14 (CAPN14), Krüppel-like factor 13 (KLF13), and EMSY have been linked with an increased risk of developing the disease [102,103,104] together with some environmental factors including diet, formula feeding, antibiotic use, proton-pump inhibitor use during childhood, cesarean births, cold climates, and indoor pollutants [105,106]. In addition, the association between EoE and autoimmunity is recently gaining increasing attention. A retrospective cohort study by Xue et al. [107] reported that autoimmune and connective tissue disorders are found in 6% of the patients with EoE, with psoriasis, psoriatic arthritis, rheumatoid arthritis, or Hashimoto’s thyroiditis being the most common concomitant diseases. Interestingly, according to the authors, EoE patients with autoimmune/connective tissue disorders show a poorer response to topical steroid treatment. Moreover, the increased levels of serum antibodies against epithelial cell adhesion molecules such as transmembrane desmoglein-3 (DSG3) and collagen XVII (NC16A) have been found in patients with EoE and therefore proposed as the biomarkers of this condition [108].

EoE typically presents with characteristic symptoms, such as vomiting, dysphagia, and food impaction. In contrast, lower digestive tract disturbances have less specific symptoms. It depends on the centrifugal progression of the eosinophilic infiltration from the mucosa (causing abdominal pain, nausea, vomiting, dyspepsia, diarrhea, malabsorption, and the subsequent hypoalbuminemia, anemia, and weight loss) toward muscular (obstruction and intestinal intussusception) and serosal layers (peritonitis, ascites, and perforation) [109]. Uncommon clinical features include hepatitis, sclerosing cholangitis, and pancreatitis [110].

Typical endoscopic findings (e.g., erythematous, friable, and ulcerated mucosa, with vertical linear furrows and strictures in the esophageal tract and pseudopolyps in the GI tract) may help in the diagnosis [111]. Owing to a significantly more extensive GI involvement than primary EGIDs, upper and lower endoscopy should be performed in patients with multisystemic HES and HES-EGID overlap [100], even when symptoms are not suggestive. Notably, due to the patchy distribution of the eosinophilic infiltrates, at least six biopsies specimens from normal and abnormal mucosa should be collected to achieve histopathologic confirmation (e.g., the marked extracellular deposition of eosinophil granule proteins or peak eosinophil counts higher than the cut-off values of ≥15 EOS/HPF in the esophagus, ≥30 EOS/HPF in the stomach or small bowel, and ≥60 EOS/HPF in the large bowel) [6,109].

To prevent more extensive GI involvement and reduce the risk of end organ damage and thromboembolic disease, prompt treatment with corticosteroids (as the first line) to reduce eosinophilia should be administered [25,112,113].

Summary statements

- After the exclusion of all the alternative causes, even in the absence of peripheral blood HE, the evidence of an eosinophilic gastrointestinal infiltration should be considered as a sort of organ-restricted HES, which may precede, in some cases, the outbreak of a multisystemic form. Hence, the patient should be provided with an attentive follow-up.
- Despite a non-optimal sensitivity, the endoscopic investigations of both the upper and lower digestive tracts may help the diagnosis. An accurate collection of biopsies should be made for histopathologic confirmation, with at least six specimens from normal and abnormal mucosa.
- Due to the risk of extensive GI involvement and thromboembolic disease, prompt management aimed at eosinophilia reduction should be provided, with corticosteroids and biologicals as viable treatment options.
- Disease remission is typically defined by a combination of endoscopic evaluation with a reduction in GI tissue eosinophils and symptomatic improvement.
- CT (computed tomography) and MRI (magnetic resonance imaging) are critical in detecting abdominal visceral involvement in EGID. Diagnosis is often difficult and is based on the symptoms, imaging findings, histological confirmation of tissue eosinophilia, and correlation with peripheral eosinophilia. Imaging is critical in identifying characteristic organ-specific findings, although imaging findings are nonspecific.

### 4.4. Heart and Vessels 

HE frequently affects the cardiovascular system and can be associated with considerable morbidity and mortality [114]. The damage to the cardiovascular system has a complex pathogenesis characterized by three phases: acute tissue necrosis, thrombosis, and fibrosis [114]. Clinical manifestations often include heart failure, intracardiac thrombosis, myocardial infarction, arrhythmias, and pericardial effusion [114,115]. At presentation, more than 60% of the patients report dyspnea as the only symptom [114,115]. However, cardiac involvement can often be asymptomatic at the onset and should be explored within HE diagnostic work-up even in the absence of suggestive manifestations.

The first assessment to be performed is the electrocardiogram (ECG), which is usually characterized by nonspecific abnormalities or normal findings. Possible findings include nonspecific T wave inversions, left atrium dilatation, ventricular hypertrophy, right anterior hemiblock, left axis deviation, premature ventricular complexes, poor R wave growth, nonspecific S-T segment anomalies, nonspecific T wave anomalies, and first-degree atrioventricular block [72,114,115].

Echocardiography is a non-invasive examination useful for evaluating the kinetics of the ventricles, valve mobility and function, areas of fibrosis, ventricular hypertrophy, and thrombi [116]. The assessments that can be performed include a two-dimensional transthoracic echocardiogram (TTE) and transesophageal echocardiogram (TEE) [116]. Echocardiography with contrast medium is useful for evaluating ventricle morphology and quantifying left ventricular hypertrophy [114,116]. In some cases, particularly during the initial stages of the disease, echocardiography may be normal. If clinical characteristics are highly suggestive of HES and significant laboratory abnormalities are found, a cardiac MRI (CMR) should be performed. CMR is more specific and sensitive than ETT and TEE in identifying ventricular thrombi [117]. In addition, CMR with gadolinium allows to establish the degree of fibrosis and whether the tissue visualized by the echocardiography is inflammatory or fibrotic [114,117].

Myocardial biopsy remains the gold standard for the evaluation of HE-related heart damage [114]. Elevated cardiac troponin T levels, indicative of myocyte degeneration, as well as NT-pro-BNP, serve as a prognostic marker [72,114,118,119], and are typically present from the disease onset, even with normal echocardiographic findings. Importantly, there is no increase in creatine kinase [72,114,118,119].

With regard to HE-related damage to the peripheral vascular system, the cases of deep vein thrombosis and arterial thrombosis have been described [114]. Although rare, patients with HE may experience arterial and venous aneurysms, with the latter being potentially fatal [114,120]. Purpura vasculitica, papules and plaques, Raynaud’s phenomenon, and acral ulcers with evolution to gangrene have also been reported [116,120,121].

Summary statements

- The damage to the cardiovascular system has a complex pathogenesis characterized by three phases: acute tissue necrosis, thrombosis, and fibrosis.
- Heart involvement is common and responsible for a relevant burden.
- ECG and echocardiography are mandatory assessments in HES patients.

### 4.5. Nervous System

HES has been associated with several neurologic manifestations due to central nervous system involvement (CNS), such as acute-onset focal neurologic deficits [122]. The major types are stroke [123,124,125,126] or encephalopathy [122,127], with other conditions including headache, demyelination, optic neuritis, ophthalmoplegia, ataxia, seizures, and spinal cord syndromes [74,81,128,129,130]. The vasculitis of the central nervous system confirmed histologically has also been described [131,132]. 

A case series and literature review by Lee et al. [122], analyzing a total of 77 cases, found that the most common types of CNS involvement were cerebrovascular disease (63.6%) and altered mental status (40.3%). The authors also reported that direct eosinophil infiltration into brain parenchyma was rare in pathologic studies [122], suggesting a major role of the embolic mechanism in the development of CNS clinical manifestations. However, the pathogenesis of CNS manifestations remains largely unresolved. Probably in HES, several factors may participate in the development of neurological manifestations, including the hypercoagulable states associated with hypereosinophilia, the cardiogenic thromboembolism due to endomyocardial fibrosis, and local thromboses due to the eosinophil-induced endothelial dysfunction of cerebral vessels [122,123,133,134]. 

Stroke-related HES is one of the better-characterized CNS manifestations in HES patients [123,124,125,126,135]. A literature review by Ono et al. [123] reported some features, such as the localization, that may help distinguish HES-related strokes from the strokes of other origins. Indeed, multiple site involvement, especially in the border zone areas, is more common in HES-related strokes. For this reason, the evaluation of multifocal brain magnetic resonance imaging (MRI) is a useful tool in HES patients with CNS involvement, identifying the border zone distribution of cerebral ischemia and other cortical areas as the most frequent imaging patterns [122]. In addition, HES-related stroke has a higher prevalence in males and may occur as the presenting symptoms in up to 73% of the cases [123]. 

HES can also present as peripheral neuropathy [122,123,132,134,136], which, on the other hand, is common in other eosinophilic disorders such as EGPA. In a recent article by Takeuchi et al. [134] the authors underlined some aspects that can help the tentative diagnosis between these two diseases, reporting that HES patients more frequently showed polyneuropathy than mononeuritis multiplex, which is dominant in anti-neutrophil cytoplasmic antibody (ANCA)-negative EGPA, and tended to show vasculitis in the peripheral nerves less frequently compared with EGPA [134].

Treatment strategies for HES neurological manifestations include steroids, cyclophosphamide, anticoagulants, and antiplatelets [123,137]. Recently, mepolizumab has also proven to be effective in a patient with HES and CNS involvement [138].

Summary statements

- HES has been associated with several neurologic manifestations due to both central and peripheral nervous system involvement.
- The most common type of manifestations in HES patients are cerebrovascular disease and polyneuropathy for the central and peripheral nervous system, respectively.
- The pathogenetic mechanisms include the hypercoagulable states associated with hypereosinophilia, the cardiogenic thromboembolism due to endomyocardial fibrosis, and local thromboses due to the eosinophil-induced endothelial dysfunction of cerebral vessels.

## 5. Treatment Options

**Figure 4:**
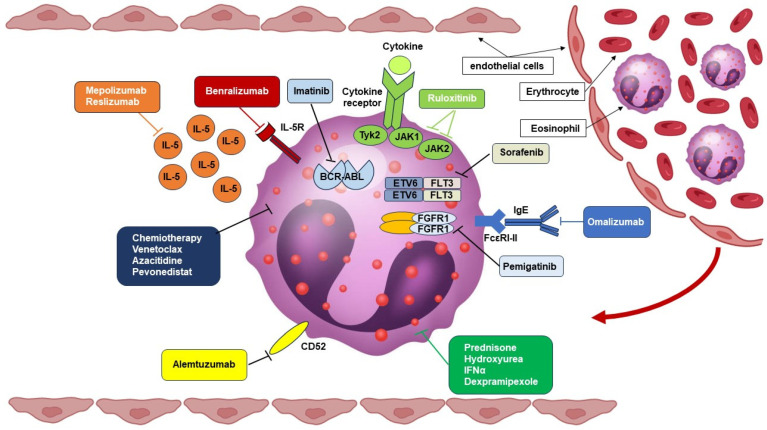
Overview of conventional, new, and developing drugs for hypereosinophilic syndrome (HES) management. ETV6, ETS variant transcription factor 6; FGFR1, fibroblast growth factor receptor 1; FLT3, Fms-related receptor tyrosine kinase 3; IL-5, interleukin 5; IL-5R, interleukin 5 receptor; IgE, immunoglobulin E; JAK2, Janus kinase 2. Created with BioRender.com.

The treatment approach to HES encompasses a number of different conventional and new drugs (Figure 4). 

**Figure 5:**
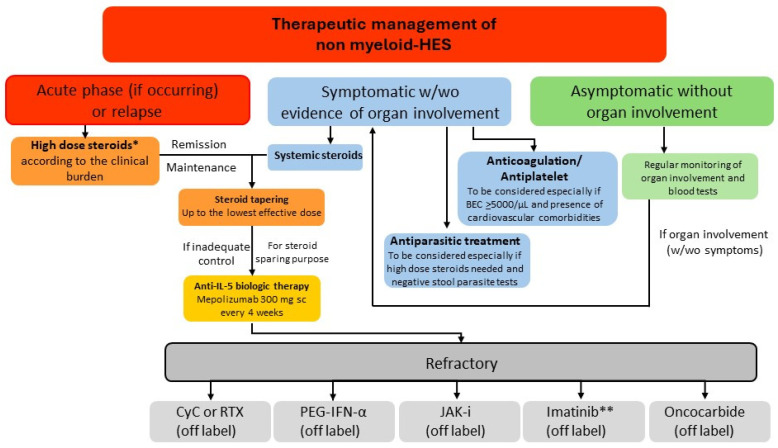
SIAAIC proposed recommendations for a hypereosinophilic syndrome (HES) therapeutic approach according to disease stage and clinical presentation. BEC, blood eosinophil count; CyC, cyclophosphamide; JAK, Janus kinase; Peg-INF-α, peginterferon-α; PDGFRA/B, platelet-derived growth factor receptor A/B; RTX, rituximab. * in the case of severe life-threatening eosinophilic respiratory, vascular, cardiac, or neurological involvement, even in the case the differential diagnosis within the HES subtypes has not been confirmed, the initial management is based on a corticosteroid therapy with 1 mg/kg/day prednisone, preceded by the intravenous pulses of methylprednisolone 5–15 mg/kg/day up to a maximum of 1000 mg for 3 days. ** especially in patients with PDGFRA/B-rearranged eosinophilic neoplasm.

The first includes treatments that were not specifically developed for HES and are characterized by a sub-optimal safety profile, especially in the long term. Very recently, the first targeted therapy for HES has been licensed and marketed. Details on the treatment armamentarium are provided below. Figure 5 summarizes the recommendations on the HES treatment approach provided by the authors according to disease stage and presentation.

### 5.1. Conventional Drugs

Corticosteroids are the first-line option for patients with idiopathic HES (HES_I_) [5,6]. The recommended dosage for adults ranges from 40 mg/day to 1 mg/kg/day of prednisone given orally; for more severe cases, 1 g of methylprednisolone per day should be used [5,139]. In children with HES_I_, a dose of 2 mg/kg/day methylprednisolone can be used as first-line treatment [5]. Treatment with corticosteroids induces a rapid reduction in the eosinophil count in most patients; however, the tapering of corticosteroids usually needs to be prolonged over months (median maintenance dose, 10 mg/day) [5,139]. Some authors have suggested that the response to corticosteroids can be predicted based on the disease subtype, with myeloid and lymphocytic HES variants having worse response compared to HES_I_, EGPA, and HES with single-organ involvement [140].

The persistence of blood eosinophilia despite the administration of corticosteroids suggests that treatment needs to be intensified with the addition of a second medication. Hydroxyurea may be used as a first-line therapy in combination with corticosteroids, or as monotherapy in non-respondents [141]. Hydroxyurea effectively controls leukocytes and eosinophils count, but there is no evidence of an influence on the natural history of HES [141]. A second-line option for patients who fail to respond to, or do not tolerate, corticosteroids and hydroxyurea is interferon-α (IFN-α) [5]. IFN-α can produce hematologic or cytogenetic remission, as well as reverse organ injury [5]. In patients with aggressive disease, bone marrow/peripheral blood allogeneic hematopoietic stem cell transplantation (HSCT) has been attempted with variable results [5]. Leukapheresis can elicit transient reductions in high leukocyte and eosinophil counts but is ineffective in the long term [5]. Anti-platelet and anticoagulant agents may be useful for preventing thromboembolism; however, a standard approach regulating their use as primary prevention in patients with HES is currently lacking [5]. Other immunosuppressive drugs such as cyclosporine, azathioprine, and methotrexate can be used in patients with HES to control the disease and as steroid-sparing drugs [141].

Currently, the use of empiric antiparasitic treatments (e.g., flubendazole or albendazole) is still a topic of debate [139]. The authors sustaining this approach underline the variable sensitivity of the parasite serology and of the available tests for the detection of parasites in stool, together with the favorable safety and low cost of these drugs, which often avoid the need for second-line investigations [139]. In addition, in patients with HES and a history of potential exposure to *Strongyloides stercoralis*, ivermectin (an anti-helminthic agent) at the dose of 200 μg/kg on day 1 followed by a second dose on day 2 or day 15 in the case of the diagnostic confirmation of strongyloidiasis should be added to corticosteroid therapy to prevent the severe reaction elicited by this pathogen in the patients receiving corticosteroids [139].

Imatinib is an effective treatment in patients with HES caused by a PDGFRA/B-rearranged eosinophilic neoplasm [5,6]. Patients with HE and the rearranged clonal marker *FIP1L1-PDGFRA* are included in the category “myeloid/lymphoid neoplasms with eosinophilia and tyrosine kinase gene fusion” and usually present an excellent response to imatinib [54,55]. Most patients achieve molecular remission with a 100 mg/day dose, while the maintenance dose may vary between 100 and 400 mg/day [142]. For patients with myeloid/lymphoid neoplasms with eosinophilia and tyrosine kinase gene fusion with eosinophilia and the *PDGFRB* rearrangement, the imatinib recommended dose is 400 mg/day for inducing remission and 100 mg/day for maintenance [55,143]. Imatinib’s safety profile in eosinophilic disorders aligns with the good tolerability seen in CML [144]. A few cases of cardiogenic shock associated with imatinib have, however, been described [59,118]. The FGFR1 inhibitor pemigatinib has recently been approved by the FDA for the treatment of patients with myeloid/lymphoid neoplasms with FGFR1 rearrangement [145]. The JAK1/JAK2 inhibitor ruxolitinib is currently under investigation in HES and primary eosinophilic disorders (NCT03801434 and NCT00044304; www.clincaltrials.gov; accessed on 1 November 2023).

Additional tyrosine kinase inhibitor therapies targeting JAK2 and FLT3 have shown promising results [56,146]. Lastly, dasatinib, an experimental anti-cancer drug designed to block the function of BCR-ABL, has been recently evaluated in several myeloproliferative disorders, including HES (NCT00255346; www.clincaltrials.gov; accessed on 1 November 2023).

Overall, while the natural history of HES associated with MLN-TK neoplasms presenting the *PDGFRA* or *PDGFRB* rearrangements has dramatically improved with the introduction of tyrosine kinase inhibitors (imatinib, in particular), neoplasms with *FGFR1*, *JAK2*, and *FLT3* fusions and *ETV6::ABL1* show variable sensitivity to the newer tyrosine kinase inhibitors [11,147,148]. In the majority of these cases, allogeneic HSCT may be the only available cure [11].

Summary statements

- For patients with idiopathic hypereosinophilic syndrome (HES), corticosteroids (e.g., prednisone 1 mg/kg) are the first option.
- Imatinib is considered a definitive treatment for PDGFRA/B-rearranged neoplasms with eosinophilia. Target therapies for patients with other molecular rearrangements such as FGFR1 inhibitors are currently under investigation.
- Additional treatment strategies include hydroxyurea, interferon-α, anti-platelet and anticoagulant agents, and bone marrow/peripheral blood stem cell allogeneic transplantation.

### 5.2. Biologic Drugs

The primary endpoint of HES treatment is clinical remission. In the case of severe organ damage, a further goal is to achieve hematologic remission (blood eosinophil count < 0.5 × 10^9^/L) to avoid relapse. Although a high initial response rate to corticosteroids is observed in most HES patients, many become refractory or develop side effects related to the long-term use of these drugs [142,149]. IL-5 plays a pivotal role in promoting eosinophils differentiation, activation, and survival [6,9]. Therefore, monoclonal antibodies (mAb) targeting this cytokine have raised increasing interest in the treatment of HES. Among these, mepolizumab, which blocks the binding of IL-5 to the α chain of the IL-5 receptor expressed on eosinophils, is the only mAb currently approved by the Food and Drug Administration (FDA) and the European Medicines Agency (EMA) with the EMA indication “as an add-on treatment for adult patients with inadequately controlled hypereosinophilic syndrome without an identifiable non-hematologic secondary cause” at the dose of 300 mg/4 weeks [150,151,152]. Initial clinical trials have shown reduced blood eosinophil counts, oral corticosteroid-sparing effect, and improvements in symptoms such as pruritus, skin lesions, nasal polyposis, and dysphagia with the intravenous administration of 750 mg mepolizumab treatment [153,154,155]. A phase III (NCT02836496), randomized, placebo-controlled trial further demonstrated the safety and efficacy of mepolizumab 300 mg administered subcutaneously every four weeks, reducing the occurrence of flares (i.e., the worsening of HES-related symptoms or blood eosinophil count requiring therapeutic escalation) in HES patients [150].

Although mepolizumab has shown its safety and efficacy in patients with HES, some issues remain to be addressed. For example, the factors predicting treatment response are still needed. Hence, the recent post hoc analysis from the same phase III study mentioned above [150] aimed to evaluate mepolizumab efficacy based on baseline blood eosinophil count and IL-5 levels [156]. Interestingly, the results showed that the drug efficacy was unrelated to baseline blood eosinophil count and IL-5 levels [156]. These results were not consistent with the previous findings in patients treated with mepolizumab 750 mg, showing that the clinical response correlated with IL-5 levels at baseline [157].

In addition, some emerging data coming from the real-life setting highlight the possibility for a personalized mepolizumab dose (300 mg/4 weeks vs. 100 mg/4 weeks) according to the disease stage (remission induction vs. remission maintenance) and patient’s profile [69,141,158].

Reslizumab is a humanized mAb that binds to circulating IL-5, preventing it from binding to IL-5R on eosinophils. Data about its use in HES patients are limited to isolated case reports and a small phase 2 trial by Klion et al. describing the use of this mAb in four HES patients [159]. Two of the patients presented a rapid decline in eosinophils after infusion accompanied by improvement in symptoms (i.e., the resolution of skin rash, mucosal ulceration, angioedema, fatigue, myalgia, and arthralgia); one patient had an initial rapid decrease in eosinophil count after baseline, and one patient did not respond to the treatment. Other case reports describing the use of reslizumab for the lymphocytic variant of HES (L-HES) have been published [160,161].

Benralizumab is a humanized mAb targeting the α subunit of IL-5R. It depletes eosinophils and their precursors by Ab-dependent cell-mediated cytotoxicity and is currently approved as an add-on maintenance treatment for adult patients with severe eosinophilic asthma. Benralizumab has shown to be effective in *PDGFRA*-negative treatment refractory HES patients in a double-blind placebo-controlled phase 2 trial [162] and in single case reports [3,163,164]. In addition, a phase 3 trial with benralizumab is currently ongoing (NCT04191304; www.clincaltrials.gov).

Finally, a randomized, double-blind, placebo-controlled study, the main objective of which is to investigate the efficacy and safety of depemokimab (a long-acting IL-5R antagonistic mAb) in adults with uncontrolled HES, has been started and is currently ongoing (NCT05334368; www.clincaltrials.gov; accessed on 1 November 2023).

Summary statements

- Patients with HES, after an initial response, may become refractory to corticosteroids or develop significant side effects related to the long-term use of these drugs.
- Mepolizumab 300 mg/4 weeks is the first biologic therapy approved for treating HES.
- The use of other biologics targeting IL-5 (reslizumab, benralizumab, and depemokimab) or different mepolizumab doses in patients with HES has also been reported.

### 5.3. Management of Specific Organ Involvement Associated with HES

If HES has been diagnosed, a systemic steroid-based approach is mandatory in order to induce the remission of the acute phase and/or prevent or limit the irreversible organ damage in the chronic phase, even in the case of specific organ involvement.

However, combining steroid treatment with a more organ-targeted therapy might be helpful in supporting specific organ functions or addressing the consequences of their impairment related to organ damage. For instance, if heart failure occurs, the use of traditional strategies, including anti-hypertensive drugs, diuretics, beta-blockers, or others according to the ongoing clinical profile, is recommended.

## 6. Recommendations for an Integrated and Multidisciplinary Approach

Generally speaking, HES shares with other rare diseases the difficult and often delayed identification of affected patients due to the limited background and awareness most physicians have about the condition. In addition, HES patients commonly present nonspecific symptoms, which further hamper the timely recognition, diagnosis confirmation, and prescribing of the most appropriate treatment [165]. 

Besides increasing the overall knowledge about the disease among all the clinicians who might potentially detect HES patients, some tools facilitating their recognition should be shared across the different levels of care, from primary care to specialists. It could be the case of a red-flags-based system tailored according to the different expertise of the healthcare professionals [165]. 

As a second requirement, once the clinical suspicion has been formulated, an effective referral is essential to shorten the patients’ journey. Dedicated healthcare pathways should link primary care to specialists, but also facilitate the specialist-to-specialist referral within the same hospital or between different hospitals characterized by diversity in terms of expertise. Structuring a multidisciplinary group, which works as a network of specialists focused on HES according to their different expertise and perspectives related to the disease, might both support the intra-hospital referral and ameliorate the standard of care provided to HES patients.

The implementation of such a model certainly depends on site-related and territorial organizational variables, but approaching HES cases through a multi-level interaction and a multidimensional discussion should guide the clinical practice even in the absence of structured healthcare pathways or before establishing them under a formal perspective.

Summary statements

- HES patients’ “hunting” needs an overall increase in the general knowledge of the disease among all the physicians potentially detecting affected patients. A red flag-based system might help in the recognition and referral of patients.
- Dedicated healthcare pathways integrating primary care and hospital centers, as well as facilitating intra-hospital referral should be consolidated.
- Besides site-related differences and organizational variables, approaching HES cases through a multi-level interaction and a multidimensional discussion is essential in clinical practice.

## 7. Discussion and Conclusions

HES encompasses a heterogeneous and complex group of different subtypes within the wider group of hypereosinophilic disorders. Despite the increasing scientific research interest recently shown in HES, several unmet needs in terms of pathobiology, phenotyping, and personalized treatment remain to be addressed. Also, disease identification remains a “by exclusion diagnosis” and the disease trajectory as well as prospective burden of non-malignant HES and, more generally, HE disorders is currently unknown. The ongoing research will hopefully generate new evidence in the field. However, in practical terms, shortening the diagnostic delay and the time to appropriate treatment approach probably represents the most urgent issue, even in light of the great impact of HES on the quality of life of the affected patients. The availability of a new targeted treatment recently licensed and marketed for idiopathic HES certainly represents a further reason to focus on the early recognition of HES patients in order to evaluate their eligibility for the first drug specifically developed for their condition.

Both the scientific unmet needs and the practical urgencies might be effectively addressed by a network approach, which allows for overcoming the limited expertise that the individual members of the physician community can acquire on HES due to its rarity. It is the first step of the “doctor’s journey” towards a better knowledge and awareness of the disease, an essential requirement for optimizing the “patient’s journey” towards the best available standard of care. 

The present document represents the first action the Italian Society of Allergy, Asthma, and Clinical Immunology (SIAAIC) has finalized within a wider project aiming to establish a collaborative national network on HES (InHES—Italian Network on HES) for patients and physicians. For patients, because InHES will provide them with a map describing the geographical distribution of referral centers for HES; for clinicians, because a structured network will facilitate data collection and sharing for scientific purposes.

The project’s first step could not preclude focusing on defining a common language and sharing with the medical community an update on the most recent advances in the field. In fact, the existing literature has been carefully reviewed in order to critically integrate the different views on the topic and derive practical recommendations on disease identification and treatment approaches.

As a major limitation of this paper, the authors acknowledge that the present statement does not provide a systematic review, and the proposed recommendations express an expert-based approach, which is not free of bias in terms of methodology. However, the document reflects the need to finally increase the community knowledge and awareness of physicians related to HES as a starting point for further and robust research, both at a national and international level. 

## Figures and Tables

**Table 1:** Classification of hypereosinophilic syndrome. ^†^ these include, among others, episodic angioedema and eosinophilia (Gleich syndrome), eosinophilic granulomatosis with polyangiitis, eosinophilia myalgia syndrome, Omenn syndrome, and the hyper-IgE syndrome. * incomplete criteria; apparent restriction to specific tissue/organs. ** peripheral hypereosinophilia in association with a defined diagnosis.

Reference	Year of Publication	HES Subclassification
Valent [6]	2023	Idiopathic Primary (neoplastic) Secondary (reactive) Familial
Shomali [5]	2022	Familial (hereditary) Primary (clonal/neoplastic) Secondary (reactive) Idiopathic
Leru [19]	2019	Secondary (reactive) Neoplastic - Myeloproliferative (M-HES) - Lymphocytic (L-HES) Familial Idiopathic Overlap * Associated ** Limited to single-organ involvement
Valent [20]	2012	Idiopathic Primary (neoplastic) Secondary (reactive) Familial Rare syndromes accompanied by HE ^†^
Fletcher [21]	2007	Reactive Clonal Idiopathic
Simon [22]	2007	Intrinsic eosinophilic disorders Extrinsic eosinophilic disorders
Klion [23]	2006	Myeloproliferative variant Lymphocytic variant Familial Undefined Overlap * Associated **
